# 浸润性黏液腺癌分子机制及临床治疗研究进展

**DOI:** 10.3779/j.issn.1009-3419.2024.106.22

**Published:** 2024-09-20

**Authors:** Minjun SHI, Bo YE

**Affiliations:** 200030 上海，上海市胸科医院，上海交通大学医学院附属胸科医院胸外科; Department of Thoratic Surgery, Shanghai Chest Hospital, Shanghai Jiao Tong University School of Medicine, Shanghai 200030, China

**Keywords:** 肺肿瘤, 浸润性黏液腺癌, 分子机制, 治疗, Lung neoplasms, Invasive mucinous adenocarcinoma, Molecule mechanism, Treatment

## Abstract

浸润性黏液腺癌（invasive mucinous adenocarcinoma, IMA）是肺腺癌的一种特殊亚型，其发病率占全部肺腺癌的2%-10%。IMA的治疗首选外科手术，传统化疗药物及靶向治疗药物对该疾病的疗效较差。与其他非黏液性肺腺癌亚型相比，IMA具有独特的预后、影像学及分子学特征。IMA的发病率极低，因此胸外科医生容易对此病缺乏认识，误诊为肺炎、结核等良性疾病。本文对IMA的影像学、临床病理特征、治疗方式及预后进行综述与讨论。

## Abstract (en)

Invasive mucinous adenocarcinoma (IMA) is a special type of lung adenocarcinoma that accounts for 2% to 10% of all lung adenocarcinoma. Surgical treatment is preferred for IMA, and traditional chemotherapy drugs and targeted therapy drugs have poor efficacy in this disease. IMA has unique prognostic, imaging and molecular features. The incidence of IMA is very low, so thoracic surgeons may lack of knowledge to the disease and misdiagnose it as benign diseases such as pneumonia and tuberculosis. This article reviews and discusses the imaging, clinicopathological features, treatment methods and prognosis of IMA.

肺癌是世界范围内因癌症导致死亡的主要病种之一，根据2020年全球癌症统计报告^[1]^，在新发癌症病例中，肺癌在男性患者中发病率及死亡率均列第一位，在女性患者中仅次于乳腺癌为发病率第二位的疾病。浸润性黏液腺癌（invasive mucinous adenocarcinoma, IMA）是肺腺癌的一种特殊亚型，其曾经被称作细支气管肺泡癌、乳头状癌、腺泡样腺癌等。在1981年世界卫生组织癌症分类中，IMA并未被分类为一种独立的亚型。IMA由Kish等^[2]^于1989年首次在病例报告中提出，并在此之后被不断研究。在2011年，国际肺癌研究学会/美国胸科学会/欧洲呼吸学会对肺癌的分类进行了一次更新，在此次更新中，学会建议停用细支气管肺泡癌的称呼，并将IMA正式分类为肺腺癌的一种独立亚型^[3]^。IMA的典型肿瘤细胞表现为细胞呈杯状或柱状并富含卵胞浆内黏蛋白^[4]^。IMA的发病率在肺癌的所有亚型中较低，占全部肺腺癌人群的2%-10%^[5]^。由于其较低的发病率，其预后情况也并未得到清晰的认识。在过去的研究^[6]^中IMA被确认与Kirsten大鼠肉瘤病毒癌基因同源物（Kirsten rat sarcoma viral oncogene homolog, KRAS）基因的突变，间变性淋巴瘤激酶（anaplastic lymphoma kinase, ALK）基因的重排以及神经调节蛋白-1（neuregulin 1, NRG1）的基因融合相关。这些基因突变位点及相关信号通路可能能够作为IMA的靶向治疗方向。相较其他的肺腺癌亚型，表皮生长因子受体（epidermal growth factor receptor, EGFR）基因的突变在IMA中较为罕见。阿法替尼作为一种对EGFR突变型非小细胞肺癌疗效较好的化疗药物，在一些NRG1基因融合的患者中表现出了较好的疗效。本文将综述IMA的分子机制及治疗等方面的研究进展。

## 1 IMA的临床病理特征

IMA的流行病学特点与其他的肺腺癌亚型大致相同。在Moon等^[5]^的回顾性研究中，其通过美国国家癌症研究所监测，流行病学和最终结果数据库中的数据比较了2000-2014年被确诊为IMA及非黏液性腺癌患者的各项指标。其中，在IMA的患者人群中，女性患者的比例相较于非黏液性腺癌要更高（1:1.45 vs 1:1.09），但在平均发病年龄上，IMA与其他肺腺癌并无明显差异（65.97 vs 66.09岁）。IMA通常好发于下肺叶并且分化良好。在发现时，IMA的分期多处在T1或T2期且多为I期肺癌^[5]^。吸烟与IMA的关系尚未明确，多项研究^[7⇓-9]^显示，吸烟均不构成IMA发病的独立危险因素。但在癌症的进展及预后研究中，Boland等^[9]^的研究显示，相较不吸烟的患者，吸烟患者的总生存率更低且无进展生存期更短。

IMA具有独特的病理表现，其典型的肿瘤细胞具有丰富的卵胞浆内黏蛋白，细胞呈柱状或杯状，一般不可见明显的细胞异型性^[3]^。IMA患者常常合并有非黏液腺癌的表现，当非黏液成分占肿瘤的比例≥10%时，应当诊断为黏液腺癌混合非黏液腺癌并明确非黏液性成分。

IMA具有独特的免疫组化特点，IMA特征性表达细胞角蛋白7（cytokeratin 7, CK7）（88%-94.7%）和CK20^[10⇓-12]^。同时甲状腺转录因子-1（thyroid transcription factor-1, TTF-1）及天冬氨酸肽酶（Napsin A）在IMA中低表达。TTF-1在75%-80%的肺腺癌中均有表达，特别是在终末呼吸单位（terminal respiratory unit, TRU）型肺腺癌中表达居多。根据Yatabe^[13]^和Sumiyoshi^[14]^等的研究结果，约有88%的TRU型肺腺癌表达TTF-1，但IMA主要为非TRU型。较TRU型肺腺癌，非TRU型肺腺癌表现出更差的预后。

## 2 IMA的影像学表现

IMA具有独特的影像学表现，其计算机断层扫描（computed tomography, CT）下的典型表现为大片浑浊影，包括肺实变及毛玻璃样阴影，通常可与大叶性肺炎表现相似。部分IMA的表现与其他类型的非小细胞肺癌相似，可表现出局部的肿块或结节^[7]^。由此可将IMA分为孤立型及肺炎型。孤立型IMA表现为CT下孤立的肿块或结节。肺炎型IMA表现为CT下无固定形状的实变影及毛玻璃样阴影，沿肺叶或肺段分布，可伴有支气管充气征^[15]^。Beck等^[7]^的研究显示，在肺炎型IMA中CT下显示出的毛玻璃样阴影由多种成分构成，包括生长在肺泡壁上的肿瘤成分，组织性肺炎，在肺泡中漂浮的包含有巨噬细胞及癌巢的黏液物质，以及纤维成分。该研究同时关注了肺炎型IMA中浑浊影的自发消退现象，即不论是否进行抗癌治疗，肺炎型IMA在多数情况下都可以出现浑浊影的减少，这与其毛玻璃影中黏液物质的减少及组织性肺炎的消退相关，因此，在评估肺炎型IMA的疗效及疾病进展时，应谨慎看待浑浊影的减少。多项研究^[7,15,16]^显示，肺炎型IMA病理分期较之孤立型IMA更高，更易在切除术后出现复发及转移，预后更差，5年无病生存率（disease-free survival, DFS）为64.7% vs 0%。有研究^[16]^关注了IMA的正电子发射断层显像（positron emission tomography, PET）/CT表现，IMA的平均最大标准摄取值（standardized uptake value max, SUVmax）是3.0，展示出临床症状的病例与未展示出临床表现的病例的SUVmax平均值分别为5.6和2.3。SUVmax值与肿瘤大小之间存在显著的关联性。对于实性结节/肿块型IMA，更高的SUVmax值出现在有临床症状或晚期的患者中。具有恶性形态体征或较大尺寸的病变也同样表现出较大的SUVmax。

## 3 IMA的分子特征

IMA的发病与KRAS基因的突变、NRG1的融合与ALK重排相关。

### 3.1 KRAS突变

KRAS突变是最常见的IMA致病突变（占发病患者的63%-90%），并且常常与其他突变一同发生。在所有的KRAS突变的IMA中，最常见的点突变为G12D、G12V和G12C，这三个位点的突变约占全部病例的40%^[17]^。Boland等^[9]^的研究分析了760例肺腺癌的患者并从中筛选了57例IMA患者及54例混合黏液腺癌的患者。其中，76%的IMA患者及68%的混合黏液腺癌患者表现出了KRAS突变，并且有38%的KRAS突变患者合并了其他基因的突变，其中占比最高的为混合丝氨酸/苏氨酸激酶11（serine/threonine kinase 11, STK11）的突变型。Tsuta等^[18]^的研究也表明了类似的结果，在其针对904例进行了手术治疗的肺腺癌患者的回顾性研究中，共有33例IMA患者表现出了KRAS的突变（IMA患者总数为45例），其比例相较其他肺腺癌亚型要显著偏高。另一项针对864例肺腺癌术后患者的回顾性研究^[19]^显示，单纯IMA患者要比混合型IMA患者更易发生KRAS突变；同时，G-A替换型突变在IMA患者中最为常见。在预后方面，KRAS突变型的早期及中期非小细胞肺癌较之未突变型的长期生存率及无进展生存期要表现更差。这证明KRAS突变可能会导致IMA患者的预后变差^[20]^。

### 3.2 NRG1基因融合

NRG1基因融合是近年来发现的具有潜力的IMA分子靶点，该种融合的发生率为7%-27%^[21]^。NRG1是一种配体蛋白，是生长因子家族的一员。其结构与EGF相似，其功能为负责激活ErbB受体酪氨酸激酶^[22]^。NRG1在神经系统、心脏及乳房中具有重要的功能，同时与多个器官系统的发育和人类疾病密切相关。NRG1的融合会导致人表皮生长因子受体2及4（human epidermal growth factor receptor 2/4, HER2/HRE4）的通路被持续激活，从而引发不受控制的细胞增殖和肿瘤的形成^[23]^。NRG1基因在正常的肺组织以及多数的肺腺癌组织上不表达，但在部分IMA患者中可见到NRG1 III-b3的融合^[24]^。IMA患者中一些常见的NRG1基因融合包括CD74（最常见）、补充溶质载体家族3成员2（solute carrier family 3 member 2, SLC3A2）、囊泡相关膜蛋白2（vesicle associated membrane protein 2, VAMP2）、联癸烷4（syndecan 4, SDC4）^[17,25,26]^。 Fernandez-Cuesta等^[27]^将表达正常水平酪氨酸激酶受体2（Erb-b2 receptor tyrosine kinase 2, ERBB2）和ERBB3的H322和H1568肺癌细胞系转导为表达CD74-NRG1融合转录物的逆转录病毒。他们发现在表达CD74-NRG1的H322和H1568细胞中，p-ERBB2、p-ERBB3、p-AKT和p-S6K的水平显著高于空载体对照和表达CD74-NRG1ΔEGF的细胞（将EGF结构域截除的版本）。此外，观察到EGF样结构域与p-ERBB3和p-AKT水平升高之间存在很强的相关性。本实验证实CD74-NRG1是驱动基因，通过NRG1 III-b3 EGF样结构域的过表达诱导ERBB3磷酸化，随后激活下游PI3K-AKT通路。在该项研究中，研究者对25例KRAS和EGFR阴性的肺腺癌患者进行了测序，结果表明，其中15例患者为IMA患者，在这些IMA患者中，有4例表现出了CD74-NRG1融合并且未检测出包括EGFR/KRAS/ERBB2/ALK/ROS1等已知致癌突变。研究者推测CD74-NRG1融合可能与已知的致癌突变互相独立。研究者^[27]^同时证明了CD73-NRG1通过ERBB2-ERBB3异二聚体信号激活PI3K/AKT通路来诱导癌症生长。基于已知可用的针对ERBB2、ERBB3和其下游通路的药物，CD74-NRG1融合可能能作为未来IMA治疗的新靶点。Shin等^[26]^着重研究了SLC3A2-NRG1融合的IMA，在该项研究中总共59例IMA患者中有16例患者有NRG1的融合，其中13例为SLC3A2-NRG1融合，研究者证明了SLC3A2-NRG1融合增加了ERBB2-ERBB3的磷酸化和异质双链的形成，并通过旁分泌信号通路激活了下游的PI3K/AKT/mTOR通路，从而增加了肺癌细胞的生长和增殖的可能性；同时，在预后研究中，含有NRG1融合的患者预后要显著差于不含NRG1融合的患者。该研究表明，SLC3A2-NRG1融合是IMA的驱动因素，并会导致不良的预后结果。综上所述，NRG1融合是一个重要的驱动基因，并且可能成为潜在的IMA治疗靶点^[26]^。

### 3.3 ALK重排

ALK重排可见于部分IMA患者中，在Tsuta等^[18]^的研究中，有2.2%的IMA患者表现出了ALK重排。在Qu等^[28]^的研究中，73例IMA患者中有25例检测到了ALK重排。

## 4 IMA的治疗

作为肺腺癌的一种亚型，IMA的治疗方案与其他腺癌的治疗相似。IMA的治疗应根据其分期进行选择。对于I、II期及部分III期的IMA患者，外科手术根治性切除仍是首选的治疗方案。对于不可切除的III及IV期IMA患者，治疗应在明确患者驱动基因的突变状态并进行美国东部肿瘤协作组（Eastern Cooperative Oncology Group, ECOG）体能状态（performance status, PS）评分的基础上，选择适合患者的全身治疗方案。

IMA主要发病于肺下叶，常表现为多灶性实变和肺叶之间或胸膜的转移^[29]^。多数外科手术方式采用肺叶切除术同时行淋巴结清扫，少数患者需行肺全切^[8]^。Matsui等^[8]^的研究比较了可手术治疗的IMA患者与非IMA患者的各项指标，IMA患者及非IMA患者在接受手术前的肿瘤直径平均值分别为（37±23）mm及（30±14）mm，多数IMA患者接受了肺叶切除术，相较非IMA患者，IMA患者的淋巴结转移较少（4% vs 24%），术后的肺内复发率较高（84% vs 31%）。

部分患者在术后选择进行以铂类为主的术后化疗，但多项研究^[29,30]^表明，是否进行术后化疗对于IMA患者的总生存率及DFS并无显著提升。因此，IMA患者的术后辅助治疗仍需进一步的研究。

对于没有手术指征的IMA患者，系统性治疗应在遵循一般肺腺癌的治疗方案基础上，针对患者的驱动基因突变情况进行选择。

对于IV期IMA患者一般选择以药物治疗为主的治疗方案。Jang等^[31]^的研究关注了进展期及出现转移的IMA患者的治疗方案，其中，常规的化疗方案是采取铂类联合培美曲塞或铂类联合吉西他滨，该项研究显示只进行化疗对IMA的总生存期没有明显的改善。

在靶向治疗方面，由于IMA少见EGFR突变，EGFR酪氨酸激酶抑制剂（tyrosine kinase inhibitors, TKIs）在IMA中的应用较为有限，而IMA常见的KRAS突变，目前仍未有在国内获批上市的相关药物。索托拉西布作为一种针对KRAS pG12C突变点位的特异性小分子抑制剂，近年来已在美国通过I及II期临床试验^[32]^，在该组126例患者的非小细胞肺癌队列中，37.1%的患者观察到客观缓解，客观缓解的中位持续时间为11.1个月，中位无进展生存期为6.8个月，中位总生存期为12.5个月。此外，在大多数患者中可观察到肿瘤缩小及肿瘤进展减缓^[32]^。

尽管EGFR突变少见于IMA患者，但对于可见EGFR突变的IMA患者，EGFR-TKIs仍然是其一线的靶向治疗方案。值得关注的是，多例病例报告^[22,33,34]^表明，阿法替尼对于NRG1融合的肺腺癌治疗是有效果的。Gay等^[22]^报告了2例使用阿法替尼治疗的NRG1融合的肺腺癌患者，其无进展生存期分别为12和10个月。Cheema等^[33]^同样报告了1例62岁从未吸烟的亚洲女性患者，肺部肿块的穿刺病理显示为IMA，其基因表现为CD74-NRG1融合。该患者接受了阿法替尼（40 mg qd）治疗，在治疗过程中影像学结果显示肿瘤进展减缓，疗效持续了26周。现有的研究^[22,33,34]^表明阿法替尼对于NRG1融合的肺腺癌治疗是有益处的，但仍需更大的队列研究来确证其疗效。

免疫检查点治疗是近期系统性治疗的热点方向，其在多种癌症中的疗效在近年中已被确认。其中，关键的免疫检查点蛋白为程序性死亡受体1（programmed cell death 1, PD-1）及细胞程序性死亡配体1（programmed cell death ligand 1, PD-L1）两种。在肺腺癌尤其是IMA中，PD-L1的阳性率相对较低，在Miyazawa等^[35]^的研究中，90例非小细胞肺癌患者的肿瘤组织中仅有8例为PD-L1阳性，并且检测的IMA样本中未发现PD-L1阳性病例。在Shimizu等^[36]^的研究中，25例IMA患者检测出了非常低的PD-L1表达。尽管如此，有研究^[31]^显示在传统化疗的基础上使用纳武利尤单抗或阿替利珠单抗进行免疫治疗对进展期及复发的IMA患者的总生存期可有显著的提升。

## 5 IMA的预后

由于IMA的低发病率，IMA患者的生存数据在每项研究中都十分有限，因此，IMA的预后尚未明确。在Yoshizawa^[37]^进行的回顾性研究中，对514例I期病变患者进行了Kaplan-Meier和Cox回归分析，基于DFS，将患者分为低、中、高危3个总体预后组。其中，IMA以及混合非黏液癌的IMA的5年无病生存率为76%，在各种肺腺癌的亚型中处于高危预后组。Russell等^[38]^的研究也表明，IMA是预后不良的肺腺癌病理亚型。不同表型的IMA也有不同的预后结果。从CT影像学分类上看，肺炎型IMA被认为是IMA预后不良的独立危险因素，这可能与肺炎型IMA被误认为是难治性肺炎从而长期使用抗生素效果不佳有关，同时，肺炎型IMA在手术切除后更易出现肺内复发情况，这可能也是造成其预后不良的原因之一。Lin等^[39]^的研究表明，腺泡为主型IMA淋巴结转移的发生率要远高于贴壁为主型IMA，生存分析显示腺泡为主型IMA的预后情况更差。Shimizu等^[36]^观察到肺炎型IMA与不良预后有显著的关联性，同时肺炎型IMA也会导致更高的复发率。综上所述，IMA患者的预后情况尚不明确。现有的研究^[36⇓⇓-39]^表明，IMA可能与不良预后有关，不同种类的IMA之间存在预后差异，但各项研究的样本量均较为有限，明确IMA的预后结果仍需要通过大样本量的队列研究做进一步分析。

## 6 总结

IMA作为一种肺腺癌的特殊亚型，关于其的研究相对有限。其流行病学特点与其他肺腺癌亚型相似，但其发病率在所有肺腺癌中相对较低。IMA的诊断可根据影像学及病理结果来确诊并分类。KRAS突变是IMA最为常见的基因突变，腺癌常见的EGFR突变在IMA中并不常见，NRG1融合是近年新发现的IMA驱动基因，其可能成为潜在的治疗靶点。外科手术是IMA的首选治疗方法，铂类为主的术后化疗效果对IMA患者疗效不明显。对于没有手术指征的IMA患者，系统性治疗是主要的治疗方式，传统以铂类为主的化疗对于IMA疗效并不显著，针对KRAS突变的靶向药物索托拉西布在近年已完成II期临床试验并在治疗具有相关突变的非小细胞肺癌上表现出了疗效。第二代EGFR-TKIs药物阿法替尼在多项研究中被报告对于表达NRG1融合的IMA有疗效，但仍需大样本队列研究来确认其疗效。IMA患者的预后尚存争议，多项研究认为IMA与较差的预后相关，但各项研究样本量均较小，得出可信服的结论仍需进一步研究。
